# Beyond Phytosterol Profiling in Greek Olive Oil: Biological Variability, Regulatory Interpretation and the Near-Threshold Sterolic Space

**DOI:** 10.3390/molecules31183170

**Published:** 2026-09-09

**Authors:** Ioanna Dialyna, Emmanouil Trantas, Filippos Ververidis

**Affiliations:** 1 Analysis and Optimization of Olive Oil Quality Group, Laboratory of Biotechnological Applications and Plant Protection (LBAP), Department of Agriculture, School of Agricultural Sciences, Hellenic Mediterranean University (HMU), Estavromenos, GR71410 Heraklion, Greece; dialyna@hmu.gr; 2 Institute of Agri-Food and Life Sciences, University Research and Innovation Center (URIC), Hellenic Mediterranean University (HMU), Estavromenos, GR71410 Heraklion, Greece

**Keywords:** Koroneiki cultivar, total sterols, campesterol, measurement uncertainty, guard band, food authenticity, GC-FID, chemometrics

## Abstract

Phytosterols are key minor constituents of olive oil, contributing not only to its nutritional and bioactive properties but also to authenticity assessment, quality control, and regulatory classification. In Greek olive oils, particularly extra virgin olive oils from the dominant Koroneiki cultivar, sterolic composition is a critical yet complex marker, as total-sterol content and specific sterol fractions may naturally approach regulatory thresholds without indicating adulteration or quality loss. This review critically synthesizes current knowledge on phytosterol profiling in Greek olive oil, emphasizing sterolic composition, analytical methodology, stability, and the principal biological, geographical, technological, and methodological factors governing sterolic variability. Particular attention is given to the sterolic fingerprint of the Greek cultivars for which comparable data exist—principally Koroneiki, with Mastoides and Lianolia Kerkyras as comparators—dominated by β-sitosterol, Δ^5^-avenasterol, campesterol, stigmasterol, and Δ^7^-sterols, and to cultivar-dependent variation that may influence regulatory interpretation. The review further evaluates the official GC-FID analytical methodology, the complementary role of GC-MS and chemometric approaches, and the limitations of interpreting sterolic composition solely through fixed regulatory thresholds. It proposes a context-dependent framework for interpreting phytosterol composition in authentic Greek olive oils and introduces the concept of the *Near-Threshold Sterolic Space*, describing the natural clustering of authentic oils close to regulatory decision limits as a consequence of cultivar- and terroir-driven biological variability. Overall, phytosterol interpretation should evolve from a purely threshold-based regulatory exercise toward a biologically informed, method-aware and cultivar-sensitive framework that better reflects the natural complexity of authentic Koroneiki, and the other Greek cultivars examined here.

## 1. Introduction

Virgin and extra virgin olive oils (VOO/EVOO) are among the most extensively characterized edible oils, owing to their nutritional and health significance, economic importance, and the stringent regulatory framework governing their quality and authenticity [1,2,3]. Greek olive oil, marketed mainly as VOO/EVOO, is a major agricultural product with a strong cultivar- and region-linked identity across mainland and insular production areas [4,5]. Among its minor bioactive components, phytosterols are a structurally distinct class of plant-derived sterols resembling cholesterol, found in the unsaponifiable fraction [6,7], and are routinely used as quality and authenticity markers; they remain determinant in the regulatory evaluation of VOO/EVOO [8].

The predominant olive-oil sterols—β-sitosterol, campesterol, stigmasterol and Δ^5^-avenasterol—form a characteristic fingerprint used to both screen for adulteration with seed or refined oils and to assess compliance with compositional limits (e.g., total sterols, campesterol, Δ^7^-sterols, erythrodiol and uvaol) [9].

Beyond this analytical role, phytosterols contribute to the nutritional value of olive oil, chiefly through their cholesterol-lowering activity and, to a lesser extent, reported anti-inflammatory and antioxidant properties [10,11]. These effects are best considered not in isolation, but as part of the broader bioactive minor fraction that also includes phenolic compounds, tocopherols, squalene, and triterpenic compounds [12,13]. Within the context of olive oil, however, the principal importance of phytosterols remains their role as robust compositional markers for authenticity, quality control and regulatory classification.

During the last decade, studies on Greek olive oils have demonstrated that phytosterol composition is shaped by complex interactions among cultivar, geographical origin, environmental conditions, fruit maturity and processing practices. Rather than representing fixed compositional values, sterolic profiles emerge as biologically dynamic fingerprints reflecting both genetic background and terroir. This is particularly evident for the dominant Greek cultivar Koroneiki, whose authentic oils frequently exhibit total-sterol contents and individual-sterol fractions approaching regulatory limits while fully complying with all other quality criteria. These observations raise important questions regarding the interpretation of sterolic composition within current regulatory frameworks [14].

Targeted data from Messinia and “Kalamata PDO” oils, combining sterols and triterpenic diols with fatty acids and waxes, indicate occasional deviations from regulatory thresholds with direct implications for compliance and PDO certification. Comparison between Greek cultivars further shows that both total-sterol content and individual-sterol composition shift with variety, implying that sterolic data must be interpreted against cultivar-specific baselines rather than generic Mediterranean averages [15,16].

Greek studies also employ phytosterols within multiparametric models: combined with fatty acids and chemometrics for monovarietal EVOO discrimination (e.g., Koroneiki versus Lianolia Kerkyras) in Western and North-western Greece [16], and more recently, integrated with machine learning (ML) on Messinian oils to predict sensory characteristics from chemical composition—linking sterolic composition and functional quality at the product level [17].

Despite these advances, a critical interpretative gap remains: existing studies have largely described sterolic composition, with comparatively little attention to how biological variability, analytical methodology and regulatory thresholds interact during authenticity assessment. As a result, values close to regulatory decision limits are often interpreted without adequate consideration of cultivar-specific baselines, methodological uncertainty or complementary compositional evidence—with tangible consequences for compliance decisions [18].

Accordingly, this review moves beyond a descriptive synthesis and proposes a context-dependent interpretative framework for evaluating sterolic profiles in Greek olive oil [15,16,17,18,19,20,21]. Integrating recent evidence on sterolic composition, analytical methodology, biological variability, stability and regulatory criteria [8,20,22,23,24], it introduces the concept of the *Near-Threshold Sterolic Space*—the natural clustering of authentic Greek olive oils close to regulatory decision limits. Rather than advocating alternative thresholds, the framework offers a biologically informed, method-aware approach to interpreting sterolic data within the existing regulatory context.

The review organizes recent Greek and international evidence into a coherent, method-aware narrative intended for researchers, quality-control laboratories, regulatory authorities and other stakeholders in Greek olive-oil quality and authenticity. Although extra virgin olive oil is the primary focus of available Greek phytosterol datasets, the broader term “Greek olive oil” is used throughout to reflect the analytical and regulatory relevance of sterols across olive-oil categories, while emphasizing EVOO as the main source of compositional and interpretative evidence [22,25].

**Literature search strategy and study selection.** To assemble the evidence base of this structured narrative review transparently and to limit selection bias, a documented literature search was performed in Scopus and complemented by backward and forward citation tracking (“snowballing”) of the key retrieved articles and of the official EU/IOC method and regulatory documents. The search combined three concept blocks: (i) sterols/phytosterols, (ii) olive oil, and (iii) Greek origin or cultivar, with the cultivar terms additionally retrieving studies of the same cultivars grown elsewhere, which were used for methodological and contextual comparison; the full search string is given in Supplementary Table S2. The search covered January 2010 to August 2026, was restricted to peer-reviewed articles and reviews published in English and was run on 4 August 2026. Studies were eligible if they reported the sterol or phytosterol composition or content of olive oil, analytical methods for their determination, factors affecting them, or their regulatory and authenticity interpretation; studies were excluded if they concerned non-olive matrices, addressed sterols solely in a nutritional or clinical context without compositional data, or lacked extractable sterol data; conference contributions were not retained as primary evidence, except for a small number of peer-reviewed proceedings reporting primary cultivar-specific sterol data central to this review (e.g., [21,26]). Greek cultivar/origin studies constitute the primary evidence base (principally Koroneiki, with Mastoides and Lianolia Kerkyras as comparators; [15,16,18]), while studies of the same cultivars grown outside Greece were used for context. The search identified 37 records with no duplicates; after screening against the eligibility criteria, five were excluded, and 32 were retained as the empirical evidence base, with additional methodological, regulatory and foundational sources incorporated through citation tracking.

## 2. Phytosterols Composition in Greek Olive Oils

### 2.1. Structural and Biological Characteristics of Phytosterols

Phytosterols are triterpenoid-derived isoprenoid lipids that constitute the principal sterols of plant cell membranes, synthesized via the cytosolic mevalonate pathway. They regulate membrane organization, permeability, and fluidity [27,28], as well as membrane-associated metabolic processes. Beyond this structural role, they have become key compositional markers for the authentication and regulatory assessment of edible vegetable oils, particularly olive oil.

The term “phytosterol” originates from the Greek words “phytò” (plant) and “stereòs” (solid), reflecting their rigid structural nature in biological systems, followed by the suffix “-ol”, which denotes the presence of a polar hydroxyl group in their chemical structure [29]. To date, over 250 distinct sterols and sterol conjugates have been identified in plants, including free sterols, sterol esters, sterol glucosides, and acylated sterol glucosides; this structural diversity underlies the wide range of biological functions that sterols perform in plant physiology [30].

Sterols are amphiphilic compounds consisting of three principal structural components: a polar hydroxyl group, a rigid tetracyclic steroid nucleus, and a non-polar alkyl side chain. In contrast to animals and fungi, whose cell membranes predominantly contain cholesterol and ergosterol, respectively, plant membranes are characterized by a high structural diversity of sterol species [31]. According to IUPAC, phytosterols exhibit a polycyclic structure, with the carbon skeleton of most molecules consisting of 28 or 29 carbon atoms, with one or two double bonds between carbon atoms. They constitute a subgroup of steroids characterized by a β-oriented hydroxyl group at the C-3 position and a tetracyclic carbon framework derived from the cholestane skeleton, closely analogous to cholesterols; they differ primarily in the alkyl substituents of the side chain [13]. Phytosterols (the plant-derived sterols) are further distinguished by alkyl substitutions at the C-24 position of the side chain, a feature that differentiates them structurally from cholesterol and largely underlies their functional diversity in plant membranes [32]. This diversity mainly arises from variations in the alkyl side chain, including differences in length, degree of unsaturation, and structural configuration, leading to distinct steric conformations among sterols. In plants, β-sitosterol, stigmasterol, and campesterol represent the most abundant sterols at the plasma membrane. Sterol molecules bearing a 3β-hydroxyl group are classified as free sterols (FSs), whereas chemical modifications of this functional group give rise to sterol conjugates. Phytosterol conjugates are primarily represented by three major classes: steryl esters (SEs), steryl glycosides (SGs), and acyl steryl glycosides (ASGs) [33]. In order to facilitate the structural characterization and systematic classification of phytosterols, they are also grouped into three major categories based on the number of methyl groups at the C-4 position: sterols bearing two methyl groups (4,4-dimethylsterols), sterols bearing one methyl group (4-methylsterols), and sterols lacking methyl groups (4-desmethylsterols) [34]. Recent advances further show that plant sterols are not passive membrane constituents, but active regulators of plasma membrane organization, protein dynamics, signalling, development, and stress response; notably, their role in forming membrane domains is particularly relevant, since plant sterols participate in the formation of membrane nanodomains involved in signal transduction, trafficking and plant–environment interactions [30,33,35,36].

Consequently, the phytosterol profile of olive oil should be regarded as the biochemical fingerprint of sterol metabolism in the olive fruit, primarily determined by genetic background and subsequently modulated by environmental conditions, ripening stage, and physiological responses to abiotic and biotic stress.

Edible vegetable oils are among the main dietary sources of phytosterols, though total content and individual-sterol distribution vary substantially among oil types. Olive oil contains a lower total phytosterol concentration than many seed oils, yet its sterolic composition is considerably more distinctive, providing one of the most informative fingerprints for authenticity assessment and regulatory classification. Representative oils used in authenticity and adulteration studies are compared with olive oil in Table 1 [8,20,37].

**Table 1:** Typical phytosterol content of selected edible vegetable oils and relevance to olive-oil profiling.

Edible Oil/Source	Typical Total Phytosterol Content (mg/100 g of Oil) *	Main Phytosterols Commonly Considered	Relevance to Olive-Oil Profiling	[Ref.]
Wheat germ oil	1700–2600	β-Sitosterol, campesterol, stigmasterol	Very high-phytosterol oil; useful as a comparative reference for phytosterol-rich edible oils.	[31,34,37]
Crude corn oil	780–1390	β-Sitosterol, campesterol, stigmasterol	High-phytosterol seed oil; relevant in authenticity studies because its sterolic profile differs from that of olive oil.	[31,34,38]
Corn germ oil	1070	β-Sitosterol, campesterol, stigmasterol	High-phytosterol oil; useful for comparison with olive-derived phytosterol fingerprints.	[31,34]
Crude rapeseed oil	680–880	β-Sitosterol, campesterol, stigmasterol	Important comparator because brassicasterol and altered campesterol levels may indicate non-olive-oil contribution.	[31,34]
Crude soybean oil	300–440	β-Sitosterol, campesterol, stigmasterol	Common edible oil with a sterolic profile distinct from olive oil; relevant in adulteration screening.	[34,38,39]
Olive oil	256–283	β-Sitosterol, Δ^5^-avenasterol, campesterol, stigmasterol	Moderate total phytosterol content but highly characteristic phytosterol fingerprint; important for authenticity and regulatory assessment.	[8,34,39]
Palm oil	70–80	β-Sitosterol, campesterol, stigmasterol	Lower total phytosterol content; distinct profile relevant in adulteration screening of olive oil.	[34,38]

**Figure 1:**
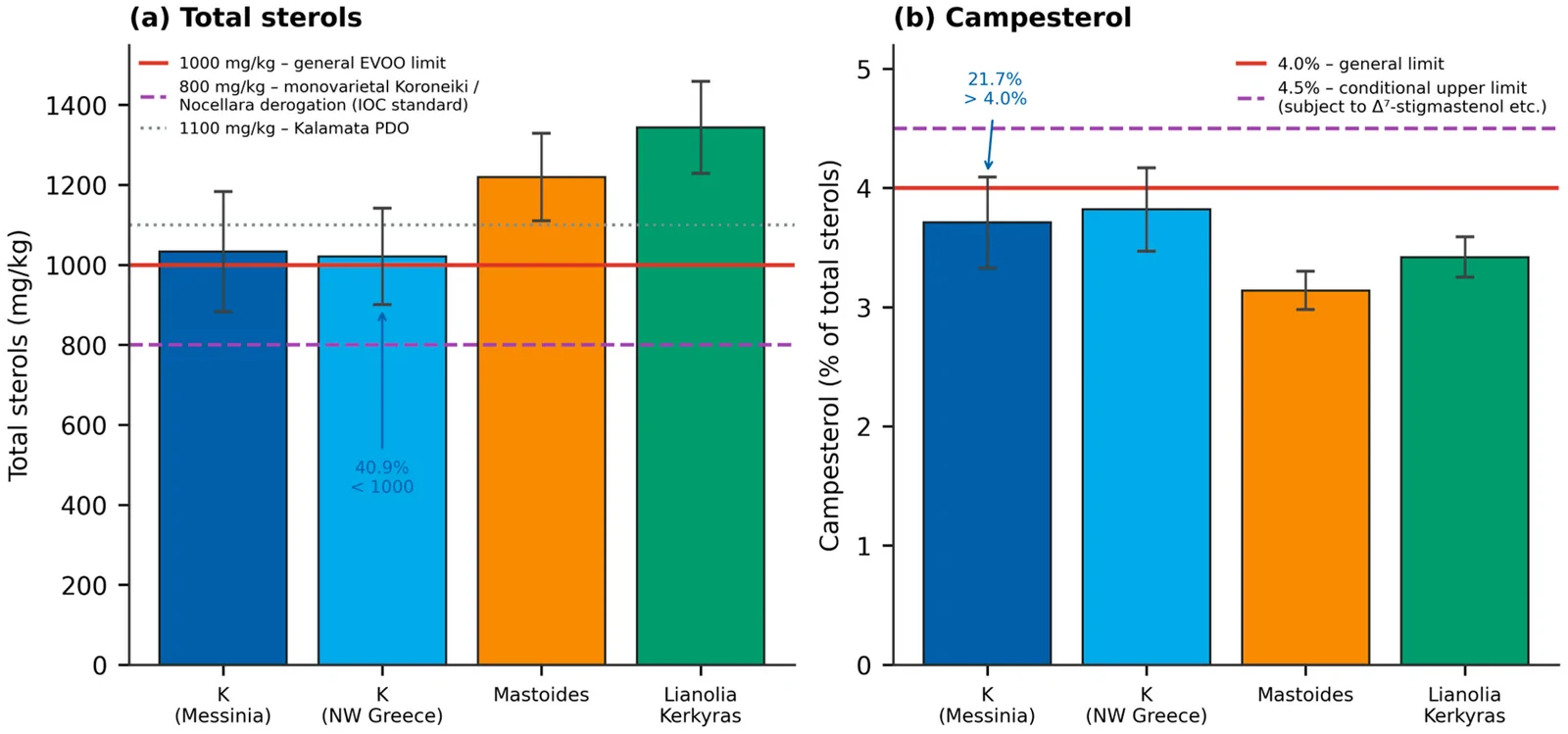
Near-threshold behaviour of the two regulated sterolic parameters in Greek monovarietal extra virgin olive oils. (**a**) Total sterols (mg/kg) and (**b**) campesterol (% of total sterols) for cv. Koroneiki (Messinia, 2014–2015, N = 69; and coastline of NW Greece, 2019–2020, N = 44), cv. Mastoides (Lakonia, 2014–2015, N = 43) and cv. Lianolia Kerkyras (NW Greece, 2019–2020, N = 60). Bars are study-reported means; error bars are ±SD; data compiled from Skiada et al. [15,16,18], with all values and sources given in Table S1. Reference lines: the general extra virgin olive-oil purity limit (1000 mg/kg total sterols; 4.0% campesterol, solid); the conditional upper campesterol limit (4.5%, subject to Δ^7^-stigmastenol and other parameters, dashed); the Kalamata PDO total-sterols limit (1100 mg/kg, dotted); and the temporary monovarietal derogation for Koroneiki/Nocellara del Belice (≥800 mg/kg, dashed; IOC Trade Standard, see Section 7). Percentages annotate the fraction of Koroneiki samples below 1000 mg/kg total sterols and above 4.0% campesterol.

**Figure 2:**
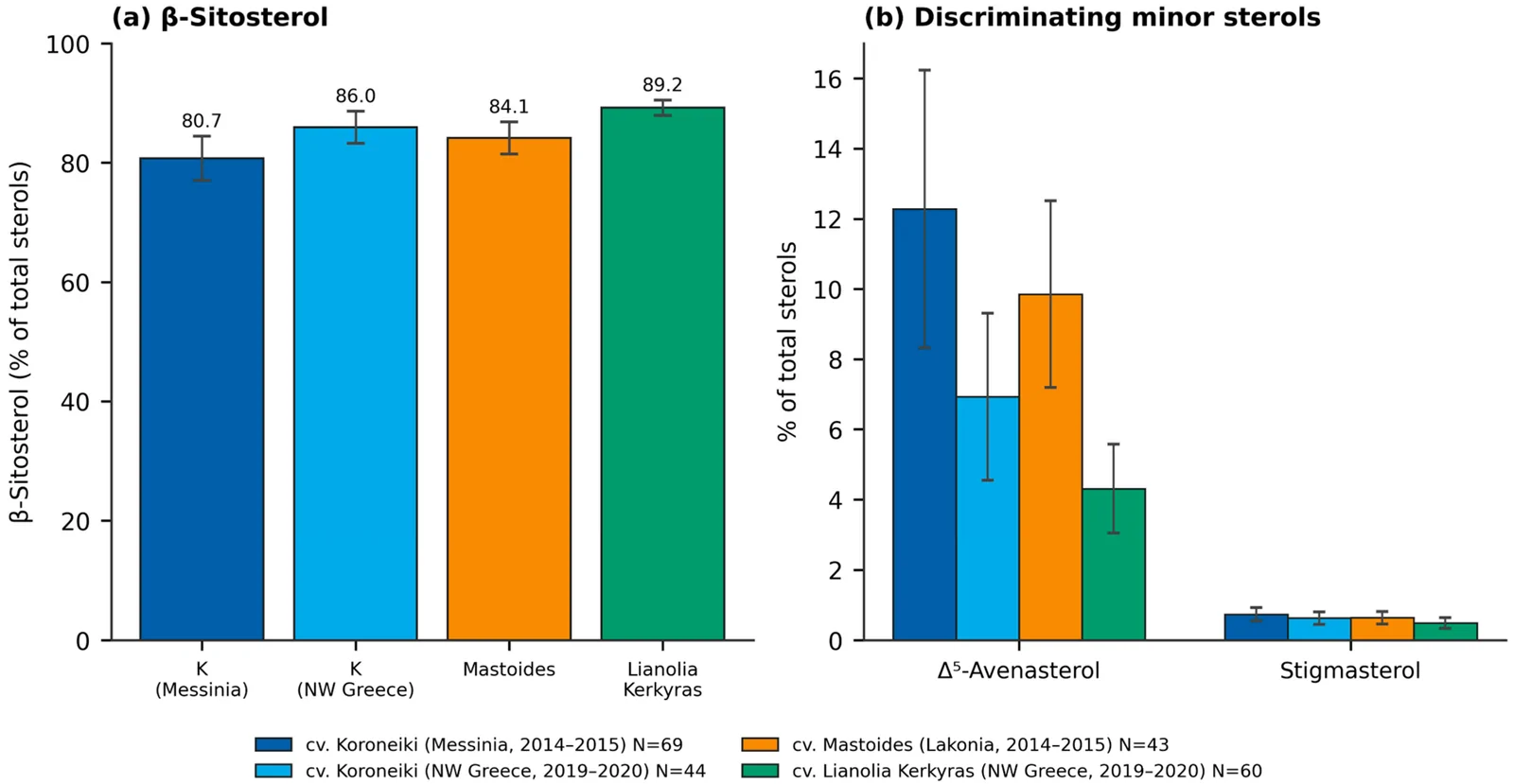
Cultivar differentiation of the sterolic profile. (**a**) β-sitosterol and (**b**) the discriminating minor sterols Δ^5^-avenasterol and stigmasterol (all as % of total sterols) for the four cultivar datasets. Bars are study-reported means; error bars are ± SD. Data compiled from Skiada et al. [15,16,18]; full source values, sample sizes, crop years, origins and methods are given in Supplementary Table S1. The inverse relationship between β-sitosterol (lowest in Koroneiki, highest in Lianolia Kerkyras) and Δ^5^-avenasterol (highest in Koroneiki) is the principal axis of cultivar separation.

* Footnote: Total phytosterol content is the sum of all sterol classes (4-desmethyl-, 4-monomethyl- and 4,4-dimethylsterols/triterpene alcohols), as reported in the cited sources. This differs from the regulated ‘total-sterols’ parameter (4-desmethylsterols only; EU/IOC limit ≥ 1000 mg/kg ≈ ≥100 mg/100 g) discussed in Section 2, Section 3, Section 4, Section 5, Section 6 and Section 7 and shown in Figure 1 and Figure 2.

Accordingly, the diagnostic value of phytosterols lies not in their absolute abundance but in their characteristic fingerprint. In Greek olive oil, this fingerprint-defined primarily by the predominance of β-sitosterol together with Δ^5^-avenasterol, campesterol, stigmasterol, and minor Δ^7^-sterols—constitutes the biochemical foundation for authenticity assessment and regulatory evaluation [8,9,20].

From an authenticity perspective, phytosterols occupy a unique position among minor constituents: unlike phenolics or volatiles, whose concentrations are strongly affected by processing and storage, sterolic composition primarily reflects intrinsic biological regulation and therefore provides a comparatively stable fingerprint—one of the reasons why sterols underpin international regulatory standards for olive-oil authenticity. This apparent stability, however, should not be mistaken for biological invariability.

### 2.2. Sterolic Composition and Cultivar-Dependent Variability in Greek Olive Oil

Across the Greek cultivars for which comparable sterol data are available, olive oils share a broadly consistent phytosterol fingerprint while showing biologically meaningful quantitative variability among cultivars and growing environments—variability that directly affects the interpretation of sterolic composition within current regulatory frameworks. The fingerprint of Greek olive oil (across EVOO, VOO and other commercial categories) consists mainly of β-sitosterol, followed by minor phytosterols such as Δ^5^-avenasterol, campesterol, stigmasterol and Δ^7^-sterols. Although phytosterols remain regulated across oil categories, the vast majority of data from the last decade concern EVOO, which forms the primary basis for the cultivar- and region-linked patterns discussed below [18].

Koroneiki oils show a strong recurring pattern across Greek datasets, with total-sterol content near the regulatory minimum of 1000 mg/kg, frequently ranging between 1020 and 1050 mg/kg [18]. Importantly, a non-negligible proportion of authentic Koroneiki oils has been reported below the 1000 mg/kg threshold despite full compliance with all other authenticity and quality criteria. This challenges the assumption that values close to regulatory thresholds necessarily indicate adulteration or compromised quality and indicates that biological variability itself must become part of the interpretative process.

Campesterol likewise sits near its 4.0% regulatory limit in Koroneiki oils (3.5–3.8%), exceeding 4.0% in a substantial proportion of samples—21.7% in the Messinia dataset (see Section 7)—particularly in Messinia and the South Peloponnese, again without any accompanying adulteration indicators [15,18]. Environmental factors may further influence where authentic oils fall relative to these limits. For cv. Koroneiki from Zakynthos, altitude has been shown to differentiate oils on the basis of quality indices, phenolic characteristics and volatile profiles [40]; that study did not measure phytosterols directly, so while it shows that pedoclimatic gradients can shift the broader compositional fingerprint of a single cultivar—and may therefore influence sterolic values near thresholds—this specific link remains to be tested rather than established, and would not, in itself, imply a quality or authenticity problem.

In the Greek olive-oil datasets reviewed here, β-sitosterol is consistently the predominant phytosterol, typically accounting for approximately 80–90% of total sterols, followed by Δ^5^-avenasterol (4–13%) and campesterol (2.5–4.7%). This ordering is preserved across the cultivars and regions for which data were available—principally Koroneiki, with Mastoides and *Lianolia Kerkyras* as comparators—consistent with a shared biochemical basis for sterol biosynthesis in olive; whether it generalizes to the many Greek cultivars still lacking sterol datasets remains to be established [6,15,41].

Phytosterol profiles in Greek olive oils present cultivar-reliant differentiations, as well. Olive oils from Mastoides exhibit higher total sterols (approximately 1200–1250 mg/kg) than the Koroneiki cultivar, harvested from the same terroir, while *Lianolia Kerkyras* shows appreciably higher total sterol values (app. 1300–1350 mg/kg), with higher β-sitosterol and meaningfully lower Δ^5^-avenasterol [15]. These observations indicate that near-threshold sterolic behaviour is cultivar-dependent rather than a universal characteristic of Greek olive oils. Data for further Greek cultivars remain comparatively sparse but are consistent with this picture; for example, the first characterization of *Mavrolia* cv. oil from Southern Peloponnese reported the same β-sitosterol-dominated profile alongside cultivar-specific quantitative differences [42].

Collectively, the evidence indicates that phytosterol composition in Greek olive oils is better represented as a continuum of overlapping cultivar-specific distributions than as isolated numerical values, so interpretation should rely on biological distributions rather than single threshold cut-offs.

As illustrated in Figure 1, Koroneiki oils occupy a lower sterolic range than Mastoides and *Lianolia Kerkyras*, positioned close to the 1000 mg/kg minimum for total sterols. Although these datasets are limited and should not be read as exhaustive cultivar bounds, the differences support interpreting near-threshold values against cultivar-specific, method-aware baselines rather than as isolated deviations.

Beyond total sterols and campesterol, the relative distribution of the major sterols adds further cultivar-discriminating information; Figure 2 compares the main sterol fractions across selected Greek cultivars.

As Figure 2 shows, cultivar differentiation appears not only in total-sterol content but in the internal distribution of individual sterols. Together, Figure 1 and Figure 2 establish that the diagnostic value of phytosterols lies in the structured organization of cultivar-specific fingerprints—the biological foundation for the interpretative approach developed in the following sections.

## 3. Analytical Methodology of Phytosterols in Greek Olive Oil: From Standardized Measurement to Context-Dependent Interpretation

Across studies of Greek olive oil over the last decade, sterolic data are usually reported as individual sterols (% of total sterols), together with total-sterol content (mg/kg) and, often, triterpenic dialcohols—a format reflecting the inclusion of sterols within the official physicochemical control framework. Most Greek datasets therefore prioritize alignment with official analytical procedures over exploratory profiling, as seen in Koroneiki-focused and PDO-relevant work and in comparative studies of Koroneiki, Mastoides, and Lianolia Kerkyras [14,15,17,20]. This consistency strengthens cross-study comparability, but also carries a key implication: when sterolic values sit close to regulatory decision limits, analytical methodology becomes an integral part of interpretation rather than a purely technical step.

### 3.1. Official Analytical Workflow: Sample Preparation and GC-FID Determination

Sterol composition in Greek olive oil is generally determined within a strict standardized framework, aligned with European Union (EU) and International Olive Council (IOC) requirements. Because phytosterols are regulated quality and authenticity parameters, most studies adopt the official EU/IOC analytical workflow, ensuring inter-laboratory comparability and regulatory assessment [17,21,24].

The analytical workflow begins with saponification of the olive-oil sample in the presence of an internal standard, commonly α-cholestanol, followed by extraction of the unsaponifiable fraction. This fraction contains sterols and triterpenic dialcohols, which are resistant to alkaline hydrolysis. Efficient recovery at this stage is essential, because incomplete hydrolysis or inefficient extraction may affect the quantitative determination of total and individual sterols [22]. The unsaponifiable fraction is then subjected to chromatographic isolation, commonly by thin-layer chromatography on silica gel plates, in order to separate the sterolic fraction from other co-extracted unsaponifiable compounds. This step reduces matrix interference before instrumental analysis and contributes substantially to the selectivity of the method.

The isolated sterols are subsequently derivatized into their corresponding trimethylsilyl ethers, usually using silylating reagents such as hexamethyldisilazane and trimethylchlorosilane. Derivatization increases volatility and improves chromatographic behaviour, enabling effective gas chromatographic separation and detection [22]. Quantitative determination is typically performed by gas chromatography coupled to flame ionization detection (GC-FID), which remains the reference method for regulatory purposes. Identification is based mainly on retention-time comparison with reference compounds, whereas quantification relies on peak areas calculated relative to the internal standard [22].

Even within this standardized workflow, individual steps—extraction efficiency, chromatographic separation, derivatization, detector response and peak integration—can influence the reported sterolic values, and these effects become especially important when authentic oils naturally occupy a near-threshold range [15,16,18,21].

### 3.2. Advanced Detection: GC-MS and Complementary Approaches

Although GC-FID remains the official reference method, gas chromatography-mass spectrometry (GC-MS) provides an important complementary approach in research, confirmatory, and metabolomic applications [43]. Because olive oil is a complex lipid matrix in which minor sterols and related unsaponifiable constituents may co-elute, GC-MS improves peak assignment through characteristic fragmentation patterns rather than retention-time matching alone—reducing misidentification, particularly for Δ^7^-sterols and other components not always fully resolved by GC-FID [8].

Beyond qualitative confirmation, GC-MS is increasingly used for targeted analysis of minor sterols and for metabolomic profiling in authenticity and traceability studies [6,44]. These applications extend beyond routine regulatory control but are valuable for interpreting borderline or atypical cases; GC-MS should therefore be seen not as a replacement for official GC-FID determination, but as a complementary tool that increases analytical confidence when sterolic profiles approach regulatory decision limits. NMR-based methods offer a further complementary route, enabling the direct quantification of total, free and esterified sterols without derivatisation [45]—a distinction of interpretive value, since the free/esterified ratio is itself an authenticity-relevant parameter.

### 3.3. Chemometrics and Data Interpretation

Olive-oil analysis increasingly incorporates chemometric and multivariate approaches for authenticity, classification, traceability, and quality assessment [43,44].

Rather than evaluating parameters in isolation, chemometric analyses multiple compositional variables simultaneously, giving a more integrated representation of chemical identity.

In Greek olive-oil research, chemometric tools have so far been applied mainly within broader compositional studies rather than in sterol-specific investigations. For example, volatile profiling combined with multivariate analysis has been used to assess the effects of cultivar and geographical origin on Greek monovarietal EVOOs, including Koroneiki, *Kolovi*, and *Adramytini*, demonstrating that chemical fingerprints can support varietal and geographical discrimination [6]. Although such studies do not focus specifically on sterols, they are relevant because they illustrate the broader transition from single-marker assessment toward multivariate interpretation in Greek olive-oil research, and individual sterols have been used among predictors in regression models for sensory attributes in Koroneiki oils [17].

Beyond Greek datasets, multi-compound approaches reinforce the value of chemometric interpretation: multi-class GC-MS profiling of the minor fraction has enabled simultaneous quantification of phenolics, triterpenic compounds, tocopherols, sterols, and free fatty acids, for varietal discrimination [46], and metabolomic/fingerprinting methods—including multivariate ^1^H-NMR fingerprinting of the unsaponifiable fraction for geographical authentication [47]—support authentication, where complex datasets require multivariate treatment [19,48].

Despite broader adoption of chemometrics in olive-oil authenticity studies, its targeted application to sterol profiling in Greek olive oil remains limited: most Greek sterol datasets still report total and individual sterols in a largely univariate, regulatory format. Integrating sterolic data with fatty acids, phenolics, volatiles, triterpenic dialcohols and sensory attributes within multivariate frameworks could substantially improve interpretation of cultivar- and region-linked variability, especially for authentic Greek oils occupying the Near-Threshold Sterolic Space.

### 3.4. Methodological Sources of Variability in Sterol Profiling: A Regulatory Limit Is Not an Extremely Clear Line

Despite the standardization of the official EU/IOC protocol, sterol determination in olive oil is not free from methodological variability, which may arise at several stages of the workflow [23,46].

One major source of variability is sample preparation, particularly saponification and extraction of the unsaponifiable fraction. Incomplete hydrolysis or inefficient extraction may affect sterol recovery and, consequently, the quantitative expression of total sterols. Although internal standards are used to correct for recovery losses, sample preparation remains a critical step in sterol analysis, especially when results are interpreted close to regulatory thresholds [23].

Chromatographic isolation of the sterolic fraction, commonly performed by thin-layer chromatography on basic silica gel plates, is also important for analytical selectivity. Variations in plate activation, solvent composition, band visualization, and band collection may influence the purity of the isolated sterolic fraction and contribute to differences in reported sterolic values [22,23].

At the instrumental level, GC conditions—column selectivity, temperature programming, resolution, detector response, and peak integration—may further affect reported sterolic composition, with GC-MS offering additional confirmation for minor or structurally related sterols (Section 3.2) [44,48].

Reporting format also influences interpretation. Individual sterols are usually expressed as percentages of total sterols, whereas total sterols are reported in mg/kg. Therefore, small absolute changes in one compound may affect the relative percentages of others, especially for regulated parameters such as campesterol. This is particularly relevant for Greek datasets in which Koroneiki oils frequently occupy a sterolic range close to regulatory decision limits [16,18].

A systematic weakness, however, is the lack of Greek studies comparing alternative or complementary workflows—parallel GC-FID/GC-MS confirmation, inter-laboratory validation, or free-versus-esterified sterol profiling—which limits any quantification of method-driven variability in Greek olive oils. Where such approaches have been validated in the broader literature (for instance, a validated SPE-GC-FID method resolving free and esterified minor compounds for authenticity assessment [49])**,** they provide a template that Greek sterol studies have yet to adopt systematically.

In sum, phytosterol determination extends beyond accurate quantification: when authentic Greek oils sit close to regulatory decision limits, methodological transparency becomes as important as the measurement itself, and reliable assessment depends on method-aware, cultivar-sensitive interpretation (developed in Section 6).

## 4. Stability of Phytosterols in Greek Olive Oil

Phytosterols are among the most chemically stable constituents of the unsaponifiable fraction, especially compared with labile components such as phenolics and pigments [50,51,52,53]. This stability underpins their use as authenticity and regulatory markers, since sterolic composition is governed mainly by intrinsic factors—cultivar, geographical origin, and ripening stage—rather than short-term post-harvest degradation. Chemical stability, however, should not be read as compositional invariability, particularly when values sit close to regulatory thresholds.

Although phytosterols exhibit considerable stability, they are not completely inert. Under oxidative conditions, particularly in the presence of oxygen, light, and elevated temperature, olive oil undergoes progressive compositional changes associated with lipid oxidation [52]. Within this oxidative environment, phytosterols may also be converted into oxidation products (oxysterols), accompanied by minor reductions in total-sterol content and subtle changes in the relative abundance of individual sterols [21]. These oxidative processes are generally slow and limited in properly preserved extra virgin olive oils and are considerably less pronounced than the degradation observed for more oxidation-sensitive constituents.

Studies on Greek olive oil consistently show that sterolic profiles are primarily cultivar- and region-dependent fingerprints rather than storage- or processing-driven variables. Major cultivars—Koroneiki, Mastoides and Lianolia Kerkyras—show highly reproducible sterolic distributions across production areas [16,17,19], and storage-related variability is expected to remain substantially smaller than the natural biological variability among cultivars and environments (Section 5.1).

To date, systematic studies of phytosterol stability and oxidation in Greek olive oils remain limited: most shelf-life work has targeted quality indices, phenolics, pigments, sensory attributes and oxidative stability, with little attention to sterol-specific degradation [8,53]. The 24-month *Kolovi* study, for example, evaluated filtration and refrigerated storage using free acidity, peroxide value, UV indices, sensory attributes, phenolics, pigments, and squalene, but developed no sterol-focused stability framework [24]. Under adverse storage conditions, phytosterols may undergo minor oxidative changes [53,54], and more pronounced thermal stress such as domestic deep-frying modifies the minor-component and sterolic profile and transfers bioactive minor components to the fried food [55]—although such culinary conditions lie outside authenticity assessment on the oil itself. These changes are generally modest relative to cultivar, ripening and terroir effects, but their interpretive weight can increase when sterols are expressed as percentages of total sterols near regulatory limits [20]. Storage-related modification should therefore be treated as a secondary layer superimposed on the primary cultivar- and terroir-driven fingerprint, not as the main source of sterolic variability.

Overall, phytosterols in Greek olive oil are chemically stable yet biologically dynamic markers. The scarcity of targeted Greek studies on sterol oxidation products, on free-versus-esterified sterols, and on storage-induced modifications marks a clear direction for future research; addressing it, alongside cultivar-specific baselines (Section 5.1), would improve authenticity assessment of oils near the **Near-Threshold Sterolic Space** (Section 6.5).

## 5. Factors Influencing Phytosterol Composition in Greek Olive Oil

### 5.1. Cultivar and Genetic Background

Among the various factors influencing phytosterol composition in olive oil, based on the available evidence, cultivar and genetic background appear to be the dominant determinants of sterolic variability (quantified below). Phytosterols are synthesized through tightly regulated biosynthetic pathways in the olive fruit, and their composition reflects genotype-specific metabolic regulation rather than random variation [20,54]. Consequently, the sterolic profile of an olive oil represents an intrinsic biochemical characteristic of each cultivar, providing the biological foundation upon which subsequent environmental and technological influences are superimposed.

Although a formal variance-components analysis across a balanced multi-factor Greek dataset is not yet available, an indicative comparison of effect sizes can be drawn from the method-harmonized datasets compiled in this review (Table S1) and is summarized in Table 2. Under a common laboratory and analytical protocol, total-sterol content differs markedly between cultivars—approximately 1021–1033 mg/kg for Koroneiki, 1220 mg/kg for Mastoides and 1344 mg/kg for *Lianolia Kerkyras*—corresponding to between-cultivar differences of roughly 190–320 mg/kg, or about 18–30% of the Koroneiki mean. In contrast, the two independent Koroneiki datasets, which differ in both geographical origin and crop year (Messinia, 2014–2015; and the coastline of NW Greece, 2019–2020), differ in mean total sterols by only ~12 mg/kg (~1%). The residual within-cultivar dispersion (standard deviation ≈ 120–150 mg/kg) is itself of the same order as the analytical expanded uncertainty of the official method (U ≈ 132 mg/kg; Section 6.5), implying that the contribution of secondary biological factors—geography, maturity, processing and storage— combined to total-sterol variance, beyond measurement uncertainty, is comparatively small. The same ordering holds for campesterol (between-cultivar range ≈ 0.68% against ≈ 0.11% for the Koroneiki region-and-year contrast). These figures are indicative rather than formal effect-size decomposition, but they consistently place cultivar as the dominant determinant, with environmental and technological factors acting as second-order modifiers of a cultivar-specific baseline.

In Greek olive oils, this genetic regulation is reflected in cultivar-specific fingerprints. Koroneiki, which dominates Greek production, is consistently associated with relatively low total sterol, frequently close to the 1000 mg/kg minimum [16,18], whereas Mastoides oils consistently contain higher total sterol than Koroneiki grown under comparable conditions, supporting genetically determined differences in sterol biosynthesis and accumulation [21].

Similarly, *Lianolia Kerkyras* oils show higher total sterols and characteristic differences in individual-sterol distribution relative to Koroneiki under the same framework [16]. Although β-sitosterol invariably dominates (≈80–90% of total sterols), followed by Δ^5^-avenasterol and campesterol, the proportional distribution differs systematically among cultivars, reflecting differences in the regulation of sterol biosynthesis rather than analytical or random variation [20]. Direct evidence for this genetic control comes from controlled-crossing experiments, in which hybridisation of a parental cultivar with Mediterranean varieties measurably shifted the sterol and triacylglycerol composition of the progeny [58].

This cultivar-dependent variability has direct implications for authenticity and regulatory interpretation: the combination of relatively low total sterols and moderate campesterol variability frequently positions authentic Koroneiki oils close to regulatory decision limits. This recurrent “near-threshold” behaviour documented across multiple independent Greek datasets should be read as a characteristic of the cultivar itself, not as evidence of adulteration or non-compliance [15,17,18]. Cultivar identity should therefore be the primary biological context for interpreting sterolic data.

Overall, genetic background is the principal driver of phytosterol composition in Greek olive oil, with between-cultivar differences substantially exceeding shifts attributable to region and crop year (Table 2). Environmental, agronomic, maturation, processing, and storage factors contribute, but generally by modifying a cultivar-specific baseline rather than replacing it—the biological basis for why authentic Greek oils may naturally occupy the **Near-Threshold Sterolic Space** (Section 6.5).

### 5.2. Geography and Terroir

Beyond genetic background, geographical origin and *terroir* are important secondary determinants of olive-oil composition. *Terroir* encompasses the combined effects of climate, soil, altitude, water availability and local agronomic practices, all of which influence olive fruit metabolism and, consequently, oil composition. In Greek EVOO, geographical origin contributes to compositional differentiation, particularly when fatty acids, tocopherols, squalene, phenolics and volatiles are evaluated collectively; direct evidence isolating terroir effects on phytosterols specifically, however, remains comparatively limited [7,40].

Within Greek production systems, geographical differentiation has been examined mainly in Koroneiki oils using integrated compositional and chemometric approaches. Oils from different regions—the Peloponnese, Crete and the Ionian Islands—show measurable compositional differences reflecting cultivar, environment, harvest practices, and local management, and should be read as modifications of an underlying cultivar-specific profile rather than alterations of sterolic identity [6,14,59].

Among terroir variables, altitude has received particular attention through its influence on temperature, solar radiation and water availability. Studies on Koroneiki oils from Zakynthos show altitude-dependent variation in quality indices, phenolic composition, pigments, antioxidant capacity, and volatile profiles; although these did not measure phytosterols directly, they support the broader concept that environmental gradients can modulate the chemical fingerprint of olive oil [40].

Climatic factors—temperature, rainfall and seasonal variability—further contribute to geographical heterogeneity, producing measurable within-cultivar differences. Sterols, together with phenolics and other minor constituents, contribute to origin discrimination through multivariate approaches [19]. Beyond Greek datasets, variety, growing region and water status each shape sterol composition—in a 297-sample Californian collection where region and drought stress contributed measurable effects [60], and across contrasting Iranian growing areas discriminated by sterol and fatty-acid profiles [61].

Soil properties and local agronomic practices further shape terroir-driven variability by influencing tree physiology and fruit metabolism. These effects have rarely been isolated in sterol-focused studies; instead, they are absorbed into broader chemometric models that evaluate geographical origin using multiple complementary markers (fatty acids, tocopherols, squalene, volatiles, phenolics, elemental composition and phytosterols) [7,62,63].

Overall, terroir appears to act mainly as a modifier of the cultivar-dependent sterolic baseline rather than as an independent determinant of phytosterol composition. Consistent with this, the distinctive sterolic behaviour of Koroneiki is not confined to Greece: monovarietal Koroneiki oils cultivated in Morocco [64,65] and Egypt [66] show comparable phytosterol characteristics despite markedly different environments, supporting an intrinsic, cultivar-driven origin of the pattern. Consistent with the indicative comparison in Section 5.1, geographical effects on total sterols are generally smaller than cultivar differences, though they gain interpretive weight for oils near regulatory limits (Section 6.5).

### 5.3. Harvest Maturity

Harvest maturity is a key biological factor in olive-oil composition, reflecting the physiological state of the fruit at oil extraction. During ripening, the biosynthesis, accumulation and transformation of lipid and minor compounds—including phytosterols—are dynamically regulated, producing measurable changes in concentration and relative distribution [19,20,67,68,69,70,71,72]. Harvest maturity should therefore be seen as a biological continuum influencing sterolic composition rather than an isolated source of variability.

Compared with plant phenolic compounds, phytosterols are generally considered less sensitive to fruit ripening. Nevertheless, several studies have demonstrated that both total-sterol concentration and the relative proportions of individual sterols may change during olive fruit development. These variations are typically gradual, biologically regulated, and strongly cultivar-dependent, reflecting developmental modulation of sterol biosynthetic pathways rather than abrupt metabolic shifts [20].

In Greek olive oils, direct evidence on sterolic evolution through maturation remains limited: most studies examined oils from a single harvest date or narrow ripening interval, particularly for Koroneiki, which is harvested comparatively early to maximize EVOO quality [18,19]. Where ripening and crop-year effects on Koroneiki sterols have been examined directly, both total sterols and individual-sterol proportions showed gradual, cultivar-constrained shifts, and seasonal variation has likewise been documented for Koroneiki and *Throumbolia* oils from Crete [56,57,73].

Although sterol-focused maturity studies are scarce, broader Greek investigations provide indirect evidence that harvest timing shifts the overall chemical profile, including the unsaponifiable fraction: changes in phenolics, oxidative stability, and volatiles with harvest timing suggest that sterol biosynthesis is likewise developmentally regulated, though less markedly than other minor constituents [19].

These maturity-related changes remain moderate and are largely superimposed on cultivar-specific characteristics; their overall influence appears substantially smaller than that of cultivar, consistent with the effect-size comparison in Section 5.1 [20].

Harvest maturity should therefore be treated as a **secondary interpretative layer**: it may contribute to the natural dispersion of sterolic values within a cultivar’s range, but it is not sufficient on its own to explain atypical or borderline profiles, which must first be read against cultivar identity [15,18].

Overall, harvest maturity is a biologically relevant but secondary modulator of phytosterol composition, to be interpreted alongside cultivar and geography rather than independently. The scarcity of cultivar-controlled, maturity-stage Greek studies is a clear knowledge gap; addressing it across representative growing regions would substantially improve our understanding of biological sterolic variability.

### 5.4. Processing Parameters

Processing parameters are an additional source of variability, though their influence on phytosterol composition appears less pronounced than that of cultivar, geographical origin, or fruit maturity (Section 5.1). Because virgin olive oil is produced exclusively by mechanical extraction, the natural sterolic composition established during fruit development is largely preserved, and phytosterols—as constituents of the unsaponifiable fraction—are generally regarded as stable throughout standard processing [20]. The cultivation regime may exert a comparable second-order influence: organic versus conventional management has been reported to modulate the minor-component and sterol profile of Koroneiki oil [57].

Among processing stages, **malaxation** is the most critical. Its conditions—temperature, duration, and oxygen exposure—affect enzymatic activity, emulsion breakdown, and oxidative reactions during oil extraction [70]. These have a well-established impact on phenolics, volatiles and oxidative stability, but their direct effect on phytosterols appears limited; prolonged malaxation or elevated temperatures may only indirectly promote minor sterolic changes through oxidation under suboptimal conditions [20,25].

**Filtration** may indirectly influence the long-term preservation of sterolic composition. It does not modify phytosterol concentrations, but by removing water droplets, suspended solids and residual enzymatic activity, it improves oxidative stability, helping preserve sterolic profiles during storage rather than affecting sterol biosynthesis directly [25].

In the Greek olive-oil sector, most studies examine high-quality EVOO produced under controlled mechanical extraction, where technological variability is limited; sterolic composition is therefore generally interpreted as reflecting the intrinsic biochemistry of the fruit rather than processing effects [18,19].

Broader compositional studies indicate that processing can contribute to overall chemical variability when combined with cultivar, terroir, maturity and storage; multivariate analyses of Greek olive oils show technological parameters shaping the compositional profile, though phytosterols rarely drive sample discrimination [19]—reinforcing that processing acts primarily as a secondary modifier of an established cultivar-dependent profile.

It is also important to distinguish mechanical extraction from **industrial refining processes**, which are applied to lower-quality olive oils but not to extra virgin olive oil. Refining may substantially alter sterolic composition through thermal degradation, oxidation, and the formation of sterol degradation products [71,72]. Although these technological treatments fall outside the production chain of authentic Greek EVOO, they clearly demonstrate that phytosterols remain chemically susceptible under sufficiently intensive processing conditions.

Overall, processing parameters should be regarded as secondary technological modifiers of phytosterol composition in Greek olive oil. Under standard extra virgin olive-oil production conditions, their influence remains limited compared with the cultivar-established baseline and its modulation by geography and fruit maturity; only under extreme processing, refining, or adverse storage do technological effects contribute appreciably to sterolic variability. These factors are summarized qualitatively in Table 3 and by indicative effect magnitude in Table 2.

## 6. Context-Dependent Interpretation of Phytosterols in Greek Olive Oil

### 6.1. Why “Threshold-Based Interpretation” Requires Compositional Context

Phytosterols are among the principal parameters used to evaluate olive-oil authenticity and quality within the official EU and IOC frameworks [25,46], which define fixed thresholds for total sterols and specific constituents, such as campesterol and apparent β-sitosterol. Although these harmonized criteria are robust for classification, their application to a biologically variable system raises interpretative challenges: phytosterol composition is not static but the outcome of interacting factors—cultivar, geographical origin, fruit maturity and growing conditions [15,18,20]. Consequently, fully compliant authentic oils may naturally approach regulatory decision limits without implying adulteration, deterioration, or reduced quality.

This issue is particularly relevant for Greek olive oils: Koroneiki, dominating national production, characteristically shows low total sterols frequently near the **1000 mg/kg** minimum, despite meeting all other requirements [15,18]. Proximity to a threshold should therefore be evaluated within the biological context set by cultivar identity, not read automatically as abnormal composition.

A similar consideration applies to individual sterols expressed as percentages of total sterols, particularly **campesterol**. Since these parameters are calculated relative to total-sterol concentration, modest changes in the overall sterolic profile may alter their reported proportions and position authentic oils closer to regulatory thresholds. This effect becomes especially relevant in cultivars or production regions characterized by naturally narrow compositional margins, where biological variability may influence regulatory interpretation without affecting authenticity [52,74,75].

Importantly, the current European regulatory framework already acknowledges, to a certain extent, the existence of biological variability. For example, although the regulatory maximum for **campesterol** in virgin and extra virgin olive oils is **4.0%**, oils presenting campesterol concentrations between **4.0% and 4.5%** may still comply with authenticity requirements provided that additional sterolic parameters—including **stigmasterol**, **Δ^7^-stigmastenol**, and all other regulated compositional criteria—remain within their specified limits. Thus, the regulatory framework itself recognizes that borderline sterolic values should not necessarily be interpreted in isolation but rather evaluated through complementary compositional evidence [76].

Collectively, identical sterolic values need not carry identical interpretative significance: the biological meaning of a value depends on its compositional context—cultivar, geographical origin, harvest maturity, analytical methodology, and the overall profile. Phytosterol interpretation should therefore move beyond a strictly threshold-based approach toward a **context-dependent framework** integrating regulatory criteria with biological and analytical evidence. This provides the scientific basis for the **Near-Threshold Sterolic Space**, in which authentic Greek olive oils may cluster close to regulatory limits without compromising authenticity or quality—a phenomenon paralleled by erythrodiol, which can naturally exceed its regulatory ceiling in authentic *Verdial* oils [77], reinforcing that regulatory exceedance is not by itself evidence of adulteration.

### 6.2. Cultivar-Dependent Interpretation

Because genetic background establishes each oil’s sterolic baseline (Section 5.1), cultivar identity is the primary context for interpreting sterolic data. In Greek oils, this is clearest for Koroneiki, whose characteristically low total sterols near the regulatory minimum are an intrinsic varietal trait, not evidence of adulteration or reduced quality [15,18]. Mastoides and Lianolia Kerkyras, by contrast, sit well above the limit with distinct individual-sterol distributions [15,16,21]. No single biological baseline therefore applies across cultivars—each has its own fingerprint within which natural variability must be judged.

Assessed without cultivar-specific baselines, authentic oils with naturally low or borderline values risk being mistaken for atypical or adulterated when they fall entirely within their cultivar’s expected range. Cultivar identity is thus the primary reference point for interpreting oils in the **Near-Threshold Sterolic Space**, with environmental, technological and analytical factors acting as modifiers of this genetic baseline (Section 6.5).

### 6.3. Geography and Environment as Modifiers

Beyond cultivar-specific baselines, geographical origin and environmental conditions are important secondary modifiers of phytosterol composition. As discussed in Section 5.2, environmental variables—climate, altitude, soil, water availability and local agronomic practices—produce measurable compositional variability even within a single cultivar [15,18,19]; these effects modulate an underlying genetic profile rather than defining sterolic identity.

In Greek olive-oil production, where Koroneiki is grown across diverse regions such as Messinia, Crete, and the Ionian Islands, geographical influences appear mainly as subtle quantitative shifts in total sterols and individual-sterol proportions rather than fundamental compositional changes. Although typically smaller than cultivar effects, these region-associated shifts become relevant when values already sit close to regulatory limits: a modest terroir-driven change in total sterols or campesterol may move an authentic oil slightly nearer a threshold and, if read without geographical context, appear atypical or suspicious when it simply reflects terroir-driven biological variability [18,19,26,59].

A key limitation of the current literature is that few studies isolate geographical effects on sterols while controlling for cultivar; in most Greek investigations, geography is assessed within broader datasets where sterols are interpreted alongside fatty acids, phenolics, tocopherols, or volatiles [78,79]. The available evidence therefore reflects the combined influences of genetic and environmental factors rather than the independent contribution of geography [52].

Geographical origin should therefore be treated as a secondary interpretative dimension: it does not redefine the cultivar-dependent sterolic identity but refines its expression by generating **region-specific compositional envelopes** within which authentic sterolic values should be judged (Section 6.5).

### 6.4. Methodological and Reporting Effects

Beyond biological and environmental factors, the interpretation of phytosterol data is shaped by analytical methodology and by how sterolic results are reported. Although the official **EU** and **IOC** methods provide a highly standardized framework for sterol determination, the analytical workflow itself contributes to the quantitative expression of sterolic parameters [22,25].

As discussed in Section 3.4, minor differences in sample preparation, chromatographic resolution, peak integration, or instrumental performance can influence the reported values of both total sterols and individual constituents, even within this validated workflow [25].

Further consideration arises from reporting conventions: total sterols are expressed in mg/kg, but individual sterols are expressed as percentages of total sterols. Because those percentages depend on the total, modest variation in total-sterol content can shift the calculated percentage of an individual sterol even when its absolute concentration is largely unchanged [23,76].

This is particularly relevant for campesterol, assessed against a fixed percentage threshold: in olive oils near the decision limit, small analytical or compositional variations may shift the reported campesterol percentage without any biologically meaningful change in sterol biosynthesis or oil authenticity. Borderline values should therefore be read within their analytical and compositional context, not as isolated numbers [18,20,80].

This is not a shortcoming of the official methods [22,23], but a reminder that any measurement reflects both the sample’s intrinsic composition and the standardized procedure used to quantify it—so reliable interpretation of borderline Koroneiki profiles requires awareness of both [15,18,25].

Methodological awareness is therefore integral to near-threshold interpretation: combined with biological and compositional evidence, it reduces the risk of overinterpreting borderline values that naturally occur within the **Near-Threshold Sterolic Space** (Section 6.5) [25].

### 6.5. The Near-Threshold Sterolic Space: An Operational, Uncertainty-Based Definition

Throughout the preceding sections, authentic Koroneiki oils have been described as sitting “close to” regulatory limits. To turn that qualitative observation into something testable—and to answer how close is close—this section defines the **Near-Threshold Sterolic Space (NTSS)** operationally, using the established language of measurement uncertainty.

**The underlying idea:** Every analytical result carries a margin of uncertainty: if the same oil samples were analyzed by several competent laboratories using the official method, their results would not be identical but would scatter within a predictable range. Consequently, a regulatory limit is not a sharp line. Around it lies a band, set by the precision of the method itself, within which a measured value cannot be reliably declared to be above or below the limit—the two possibilities are statistically indistinguishable. This band is what metrologists call a guard band, and it is the formal basis of the NTSS.

**From method precision to the guard band:** The precision of an analytical method is characterized, under ISO 5725 [81], by its reproducibility standard deviation, SD_R_—the standard deviation of results obtained on identical material in different laboratories, with different operators and equipment. SD_R_ captures the full, real-world scatter of the method. Following the widely used “top-down” approach to measurement uncertainty under ISO 21748 [82], SD_R_ may be taken directly as the standard uncertainty, *u*, of a single result. To express this at a defined level of confidence, the standard uncertainty is multiplied by a coverage factor, k; for an approximately normal distribution, k = 2 corresponds to ≈95% confidence [83]. The expanded uncertainty is therefore

U = k·SD_R_, with k = 2 (≈95% confidence).

For a regulated parameter *X* with official decision limit *L*, we define an oil as lying within the **Near-Threshold Sterolic Space** when its value falls inside the guard band.

**[L − U, L + U],** i.e., when |X − L| ≤ U. Within this band, following the Eurachem framework for compliance assessment [84], a result cannot be classified as compliant or non-compliant on that parameter alone; a confident decision requires the value to lie more than U from the limit. This is precisely the situation ILAC-G8 describes as a non-binary or conditional decision [85]. A cultivar is *characteristically NTSS-associated* with a given parameter when the regulatory limit falls within the central body of its authentic reference distribution rather than in its extreme tail—that is, when a substantial fraction of genuine oils of that cultivar is expected to fall inside the guard band.

**Figure 3:**
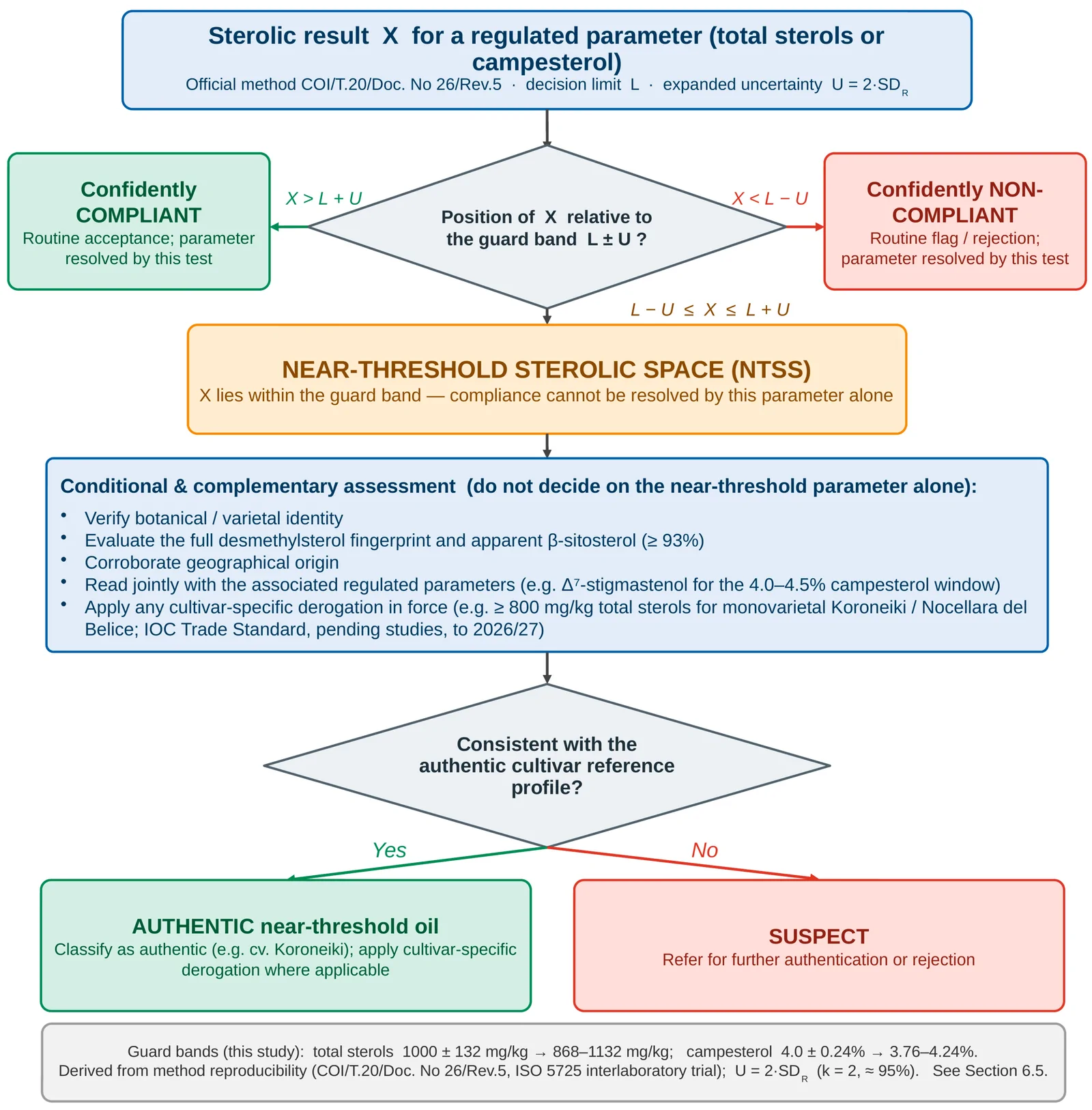
Proposed uncertainty-based decision framework for interpreting near-threshold sterolic values. For a regulated parameter *X* with official decision limit *L* and expanded measurement uncertainty *U* = 2·SD_R_ (*k* = 2, ≈95%), results lying more than *U* from the limit are resolved directly (compliant if *X* > *L* + *U*; non-compliant if *X* < *L* − *U*), whereas results within the guard band [*L* − *U*, *L* + *U*] fall in the Near-Threshold Sterolic Space (NTSS) and cannot be classified by that parameter alone. Such results are referred to as conditional and complementary assessment—botanical/varietal verification, the full desmethylsterol fingerprint and apparent β-sitosterol, geographical corroboration, joint reading of the associated regulated parameters, and any cultivar-specific derogation in force—and are classified as authentic near-threshold oils (e.g., cv. Koroneiki) only where consistent with the authentic cultivar reference profile. Guard bands derived in this study from the reproducibility of the official method [23,81]: total sterols 868–1132 mg/kg; campesterol 3.76–4.24% (Section 6.5). This scheme is an interpretative proposal of the present review; it operates within, and does not constitute, any EU or IOC regulatory criterion.

**Established concepts versus the present proposal:** The metrological components on which this definition draws—measurement uncertainty, expanded uncertainty with a coverage factor (*k* = 2, ≈ 95%), guard-banding around a decision limit, and the non-binary (conditional) classification of results falling within the guard band—are well-established in analytical metrology and conformity assessment (GUM; ISO 5725 [81]; Eurachem [84]; ILAC-G8 [85]). What the present review proposes is their integrated application to olive-oil sterol authenticity: the definition of the Near-Threshold Sterolic Space as a named, cultivar-specific interpretative construct, and the translation of a near-threshold result into an explicit decision outcome (Section 6.7; Figure 3; Table 4). The NTSS is therefore an interpretative framework proposed by the authors that operates within the official EU and IOC criteria; it does not modify, replace, or itself constitute any EU or IOC regulatory criterion.

**Applying this to Greek olive oil:** Using the reproducibility of the official sterol method COI/T.20/Doc. No 26/Rev. 5 [23], the guard bands for the two most relevant parameters are derived in Box 1. For total sterols, they span **868–1132 mg/kg** (around the 1000 mg/kg limit), and for campesterol, **3.76–4.24%** (around the 4.0% limit).

Box 1 **Deriving the Near-Threshold Sterolic Space guard bands.**

  The guard band around a regulatory limit is obtained in three steps from the reproducibility of the official method [23,86].

Step 1 **—Relative reproducibility (RSD_R_).** From the method’s ISO 5725 [81] collaborative trial, read the relative reproducibility standard deviation: **RSD_R_** ≈ 6.6% for total sterols and ≈3.0% for campesterol.
Step 2 **—Absolute standard uncertainty at the limit (SD_R_ = RSD_R_ × L)**.
  - Total sterols at L = 1000 mg/kg: **SD_R_** = 0.066 × 1000 = **66 mg/kg**.
  - Campesterol at L = 4.0%: **SD_R_** = 0.030 × 4.0 = **0.12 percentage-points**.
Step 3 **—Expanded uncertainty and guard band (U = 2·SD_R_; band = L ± U)**.
  - Total sterols: U = 2 × 66 = **132 mg/kg** → guard band **868–1132 mg/kg**.
  - Campesterol: U = 2 × 0.12 = **0.24 pp** → guard band **3.76–4.24%**.

**Parameter**

**Limit (*L*)**

**RSD_R_**

**SD_R_ = RSD_R_·*L***

**U = 2·SD_R_**

**Guard Band [*L* − U, *L* + U]**
Total sterols1000 mg/kg6.6%66 mg/kg132 mg/kg
**868–1132 mg/kg**
Campesterol4.0%3.0%0.12 pp0.24 pp
**3.76–4.24%**

  *Interpretation: a single result inside these bands cannot be reliably declared compliant or non-compliant on that parameter alone (≈95% confidence, k = 2). “pp” = percentage points of total sterols, distinguishing the campesterol unit from the relative RSD_R_*.

Overlaying the compiled Greek datasets (Table S1; Figure 1) on these bands reveals the cultivar-specific nature of the NTSS. The population means of both independent Koroneiki datasets—Messinia (1033.3 mg/kg) and the coastline of NW Greece (1020.8 mg/kg)—fall inside the total-sterols guard band, whereas cv. Mastoides (1219.6 mg/kg) and cv. Lianolia Kerkyras (1343.7 mg/kg) fall clearly above it. The same structure appears for campesterol. A complementary way to see this is to ask what proportion of each cultivar’s authentic oils is expected to fall below the 1000 mg/kg limit: approximately 41–43% for Koroneiki, but only about 2% for Mastoides and 0.1% for Lianolia Kerkyras (estimated from the cultivar means and standard deviations of Table S1, assuming approximately normal distributions). Reassuringly, the model-predicted 41–43% for Koroneiki closely matches the directly observed figures of 43.5% and 40.9% below 1000 mg/kg in the two datasets—as expected, since both derive from the same cultivar means and standard deviations; this confirms that the assumption of approximately normal cultivar distributions is reasonable for these datasets. For Koroneiki, therefore, the regulatory limit does not separate authentic from adulterated oil; it runs through the middle of the authentic distribution, so that a large share of genuine Koroneiki oil is, by construction, analytically indistinguishable from a non-compliant result.

**From a measured value to a regulatory decision:** Defining the guard band is only the first step; a laboratory still needs to translate a measured value into a feasible outcome. This mirrors a broader principle in regulatory science: an analytical result becomes useful for decision-making only when it is deliberately translated from a raw detection signal into a decision-relevant one that is decision-linked, contextual, comparable and auditable—a translation logic articulated by Parlapani et al. for omics-based food data and equally applicable here [87]. The NTSS provides exactly this translation for sterolic data. A measured value (the detection signal) is first read against the analytical guard band and the cultivar-specific baseline—making it contextual and comparable—and is then mapped to an explicit regulatory outcome under predefined criteria, with stated uncertainty and a feasible action—making it decision-linked and auditable. The resulting outcomes are summarized in Table 4.

Outcomes within the guard band are non-binary in the sense of ILAC-G8 [85]: the measured value alone does not determine compliance, and the decision is completed by cultivar identity and complementary compositional evidence (Figure 3).

**Two different sources of variability—kept separate:** It is essential to distinguish between two quantities that both place Koroneiki near the limit but for different reasons. The analytical (measurement) uncertainty, U ≈ 132 mg/kg, describes how much a measurement of a given oil could vary between laboratories; it defines the guard band. The biological variability, expressed as the between-sample standard deviation in Table S1 (≈120–150 mg/kg for Koroneiki), describes how much authentic oils themselves genuinely differ from one another; it governs the proportion falling below the limit. These must not be conflated: the guard band asks whether a measurement can be trusted to sit on one side of the line, while the biological spread asks how many genuine oils legitimately do. Notably, for Koroneiki the two are of similar magnitude—the biological SD is roughly equal to the expanded analytical uncertainty—which is exactly why near-threshold classification is so fragile for this cultivar: both measurement scatter and natural biology place authentic oils on both sides of the limit.

**A note on the precision estimate:** The collaborative trial underpinning the official method characterized materials at higher concentrations than the decision limits (1572–3181 mg/kg total sterols; 3.1–8.4% campesterol). Deriving SD_R_ at the limits therefore assumes that the relative reproducibility (RSD_R_) is approximately constant across this range—an assumption supported empirically by the near-constant trial values (RSD_R_ ≈ 6.6–6.9% for total sterols) and, if anything, conservative: the Horwitz relationship predicts that RSD_R_ tends to increase at lower concentrations [88], so the true guard band could be marginally wider, placing even more authentic Koroneiki oil within the NTSS. We state this explicitly as a limitation.

**Interpretation:** Within the NTSS, identical sterolic values may carry different interpretative significance depending on cultivar identity, geographical origin, analytical methodology and the overall compositional profile of the oil. The NTSS thus provides a conceptual bridge between biological variability and regulatory interpretation: rather than replacing EU or IOC authenticity criteria, it offers a scientifically grounded, quantitative basis for understanding why authentic Greek oils—particularly Koroneiki-based systems—may naturally occur close to regulatory decision limits, and for distinguishing genuine adulteration from the normal expression of cultivar-dependent variability. The decision rule that follows from this definition—routine pass/fail outside the guard band, and referral to conditional and complementary assessment within it—is developed in Section 6.7 and summarized in Figure 3; its emerging regulatory counterpart, the temporary ≥ 800 mg/kg derogation for monovarietal Koroneiki, is discussed in Section 7 [22,25,76].

### 6.6. Towards Holistic Interpretation

The limitations of threshold-based and single-parameter evaluation point to a more holistic approach, in which phytosterols are assessed not as isolated regulatory markers but as one component of a broader compositional fingerprint. In contemporary olive-oil research, this is already routine: multivariate and chemometric methods—principal component analysis, PLS regression, supervised classification and machine learning (ML) models—evaluate sterols together with fatty acids, triacylglycerols, phenolics, volatiles and tocopherols, and recent Greek studies have used such combinations to classify oils and predict sensory characteristics (Section 3.3) [17,44]. Within these models, sterols acquire greater biological and regulatory significance than when read in isolation.

This integration is especially valuable for oils in the Near-Threshold Sterolic Space, where it sharpens the distinction between natural variability and genuine adulteration: an oil with borderline sterolic values but fully consistent fatty acid, phenolic and volatile profiles is far better characterized as authentic than sterols alone would allow [19]. Such holistic interpretation complements rather than replaces the EU and IOC framework—regulatory thresholds remain indispensable for harmonized assessment, but their application becomes more scientifically robust when supported by cultivar-specific baselines and complementary compositional evidence (Section 6.5).

### 6.7. Practical Implications

The operational definition of the NTSS (Section 6.5) converts the qualitative observation that cv. Koroneiki sits “close to” its regulatory limits into a concrete decision problem. Because the population means of authentic Koroneiki oil fall inside the analytical guard bands of both the total-sterols limit (868–1132 mg/kg) and the campesterol limit (3.76–4.24%), a non-negligible fraction of genuine oil is, for these two parameters, analytically indistinguishable from a non-compliant result. A binary pass/fail rule applied at the nominal limits therefore does not separate authentic from adulterated Koroneiki oil; it partitions the authentic distribution itself, producing systematic false exceedances for one cultivar while leaving others unaffected.

The practical consequence is a two-tier interpretative rule. A sterolic result lying more than the expanded uncertainty U from a limit can be classified confidently by that parameter alone. A result falling within the NTSS guard band should instead trigger conditional and complementary assessment rather than a stand-alone verdict: verification of botanical/varietal identity, evaluation of the full desmethylsterol fingerprint and apparent β-sitosterol, corroboration of geographical origin, and joint reading of the associated regulated parameters (for campesterol, Δ^7^-stigmastenol and the other criteria attached to the 4.0–4.5% conditional window). Under this rule, the near-threshold values that dominate authentic Koroneiki are handled as an expected cultivar characteristic requiring corroboration, not as presumptive markers of adulteration. This is not a relaxation of the authenticity criteria but a metrologically consistent application of them, and it mirrors the direction the regulator has now taken (Section 7).

Because phytosterols remain essential regulatory markers within the EU and IOC frameworks [22,25,76], this context-dependent interpretation has practical implications across stakeholders, without replacing official protocols. In every case the framework supplements, rather than supplants, the established EU/IOC criteria for oils near decision limits: quality-control laboratories apply the official protocols and conditional-verification procedures, additionally weighing cultivar identity, analytical comparability and complementary markers to separate natural variability from genuine concern [7,17,19]; regulatory authorities gain a principled basis for cultivar-aware evaluation of borderline results; producers obtain justification for authentic oils that legitimately approach limits; and researchers are directed toward cultivar-specific reference datasets and the sterol-oxidation knowledge gaps identified in Section 6.8.

In short, the framework does not modify authenticity standards but supports their more informed application to authentic Greek oils occupying the NTSS.

### 6.8. Future Directions

Despite considerable progress in characterizing the chemistry of Greek olive oils over the last decade, phytosterol research remains comparatively underdeveloped. While volatile and phenolic profiling and authenticity marking have advanced across many Greek cultivars, sterol-specific datasets remain scarce for wide emblematic varieties, limiting the development of robust cultivar-specific fingerprints beyond Koroneiki and a few well-characterized comparators [6].

The foremost research priority is the development of comprehensive cultivar-specific sterol datasets spanning the major Greek growing regions and multiple seasons. These would capture the natural biological variability of sterolic composition, establish realistic per-cultivar baselines, and strengthen the interpretation under near-threshold conditions [14,17,18,19].

A second priority is the systematic investigation of the factors shaping sterolic composition under controlled conditions—the individual and combined effects of geography, climate, harvest maturity, processing and storage. Particular attention should go to the distinction between free and esterified sterols and to the formation of sterol oxidation products, both poorly characterized in Greek olive oils [19,51,54].

Methodological harmonization also deserves attention: although the official EU/IOC methods provide a standardized basis, comparative studies of inter-laboratory variability and of complementary platforms—GC-FID, confirmatory GC-MS and emerging high-resolution techniques—would improve data comparability and confidence in sterol-based authenticity assessment [8,21,24].

Integrating sterol profiling with chemometric, metabolomic, and multi-omics approaches is a particularly promising direction (Section 6.6): combining sterols with fatty acids, phenolics, volatiles and elemental fingerprints improves cultivar discrimination, geographical classification, and authenticity assessment [16,18,25,76,80], and machine learning (ML) approaches are expected to enhance the predictive value of such integrated datasets further.

Ultimately, phytosterol research should move beyond description toward biologically informed interpretative models; the **NTSS** proposed here offers an initial basis, whose refinement through large-scale, multidisciplinary datasets may support more accurate authenticity assessment within the existing EU and IOC standards (Section 6.5). A central limitation of the present synthesis is the narrowness of the primary Greek evidence base: the cultivar-resolved sterol data that anchor our quantitative statements derive largely from three cultivars—Koroneiki, Mastoides and *Lianolia Kerkyras*—characterized by a single ISO 17025-accredited laboratory over two crop years [15,16,18]. Conclusions concerning cultivars beyond these three, or the Greek varietal landscape as a whole, are therefore necessarily provisional, and the near-threshold behaviour formalized as the Near-Threshold Sterolic Space is demonstrated specifically for Koroneiki rather than asserted for Greek olive oils in general.

## 7. Regulatory Implications for Greek Olive Oils

The regulatory framework has recently moved in a direction that directly corroborates the NTSS analysis. A footnote to the current IOC Trade Standard (COI/T.15/NC No. 3/Rev. 22 June 2026, [25]) sets the minimum total-sterols limit for monovarietal extra virgin olive oils of the Koroneiki and Nocellara del Belice varieties at ≥800 mg/kg; the corresponding EU position was established by Council Decision (EU) 2025/1213 of 12 June 2025 [89]. Critically, this provision is explicitly conditional and temporary: it applies “pending further scientific studies,” is restricted to the named monovarietal oils (with the *Coratina* variety subsequently proposed for addition), and is limited in application to the end of the 2026/2027 crop year. It is therefore a cultivar-specific, time-limited derogation from the general purity limit, and must be distinguished from (i) the general extra virgin olive-oil limit of 1000 mg/kg total sterols and 4.0% campesterol, and (ii) the pre-existing conditional campesterol criterion (4.0–4.5%, subject to Δ^7^-stigmastenol and the other associated parameters).

Read against Section 6.5, this derogation is best understood as regulatory recognition of precisely the phenomenon the NTSS formalizes. By lowering the monovarietal Koroneiki limit to 800 mg/kg—i.e., below the lower edge of the total-sterols guard band (868 mg/kg)—the standard removes the systematic near-threshold conflict for that cultivar, conceding, in effect and “pending further scientific studies,” that authentic Koroneiki naturally occupies a sterolic space that the cultivar-blind 1000 mg/kg limit misclassifies. The NTSS framework supplies the quantitative and biological rationale for this move and generalizes it: it identifies campesterol (for which no equivalent cultivar-specific derogation yet exists, and where 21.7% of Messinian Koroneiki samples exceed 4.0%) as the next near-threshold parameter warranting the same conditional treatment, and it provides an uncertainty-based criterion for deciding when such derogations are justified. We note, however, that the derogation is provisional: it is monovarietal-only, so Koroneiki-containing blends and other cultivars remain subject to the 1000 mg/kg limit, and its status beyond the 2026/2027 crop year depends on the outcome of the further studies the standard itself invokes—studies to which a review such as the present one is intended to contribute.

Such contextual interpretation does not modify the official criteria but supports their informed application, consistent with the conditional assessment already embedded in the EU framework (Section 6.7).

Cultivar-specific reference datasets for authentic Greek olive oils would give quality-control laboratories and regulatory authorities a sound basis for evaluating borderline cases [17,19]. Notably, market surveys assessing commercial oils against EU purity criteria, including phytosterol parameters, periodically report borderline or non-conforming results [90], underscoring the practical relevance of context-aware interpretation at decision limits.

Overall, the EU/IOC regulatory framework remains scientifically robust and appropriate for olive-oil authenticity assessment. The contribution of this review is not to advocate changing regulatory limits, but to provide a biologically informed interpretative framework that supports their consistent application to authentic Greek olive oils. By integrating regulatory criteria with cultivar-specific biology and analytical context, it enhances the reliability, transparency and robustness of phytosterol-based authenticity assessment.

## 8. Conclusions and Perspectives

This review provides a critical synthesis of phytosterol composition in Greek olive oils, drawn principally from the Koroneiki cultivar with Mastoides and *Lianolia Kerkyras* as comparators and complemented by the international literature. Across this evidence, sterolic profiles are governed primarily by cultivar-specific biochemistry, with Koroneiki distinctively tending toward low total sterols and values close to regulatory decision limits [14,15,17]; geography, maturity, processing, storage and analytical methodology act as secondary modifiers of this cultivar-set baseline. Sterolic composition is therefore best understood as the outcome of interacting with biological and analytical processes rather than as a fixed compositional parameter.

The principal contribution of this review is a context-dependent interpretative framework centred on the **Near-Threshold Sterolic Space (NTSS)**—the compositional domain, defined here by analytical measurement uncertainty, in which authentic oils approach regulatory limits while remaining fully consistent with natural biological variability. The framework does not challenge, replace, or constitute EU or IOC regulatory criteria, but explains, quantitatively, why authentic Koroneiki oils may legitimately occupy this space, and it translates a near-threshold result into an explicit, auditable regulatory decision. Its logic is independently corroborated by the recent temporary ≥800 mg/kg derogation for monovarietal Koroneiki, which enacts precisely the cultivar-aware, conditional interpretation the NTSS formalizes.

Realizing this approach will require comprehensive cultivar-specific sterol datasets across the principal Greek cultivars, regions and seasons, harmonized analytical methodology, and study of free and esterified sterols and sterol oxidation products, ideally integrated with chemometric and multi-omics analyses. Beyond Greek olive oil, the NTSS may offer a transferable model wherever natural biological variability intersects with regulatory decision limits. Ultimately, phytosterols should be seen not merely as compliance markers but as biologically informative descriptors—interpreted through cultivar identity, analytical methodology and compositional context rather than through fixed thresholds alone.

## Figures and Tables

**Table 2:** Indicative comparison of the effect magnitude of the principal factors on total-sterol content in Greek olive oil.

Factor	Typical Effect on Total Sterols	Approx. Magnitude	Basis/Source
Cultivar (genetic background)	≈190–320 mg/kg between cultivars (difference in means under a common laboratory and method)	≈18–30%	Table S1; Skiada et al. [15,16,18]
Geographical origin and crop year (combined, within Koroneiki)	≈12 mg/kg between the two independent Koroneiki datasets (different region and crop year)	≈1%	Table S1; [15,18] vs. [16]
Harvest maturity/ripening	Second-order; not separately resolved above analytical uncertainty in harmonized Greek datasets		Ripening studies [56,57]
Processing and storage	Minor under standard EVOO production; phytosterols are relatively stable in the unsaponifiable fraction	-	[20,53,55]
Within-cultivar dispersion (all secondary factors + analytical, combined)	SD ≈ 120–150 mg/kg	≈12–15%	Table S1
Analytical measurement uncertainty (reference)	Expanded uncertainty U ≈ 132 mg/kg (k = 2)	≈13% ^†^	Section 6.5; IOC, COI/T.20/Doc. No 26/Rev.5 [23]

Note. Values are indicative and are drawn from the method-harmonized datasets compiled in Table S1; they represent a first-order comparison of effect magnitudes rather than a formal variance-components (ANOVA) decomposition, which would require a balanced multi-factor Greek dataset not yet available. Between-cultivar values are differences in mean total sterols determined under a common laboratory and analytical protocol. Relative magnitudes (%) are expressed with respect to the mean Koroneiki total-sterol content (≈1027 mg/kg). The within-cultivar dispersion (SD ≈ 120–150 mg/kg) is of the same order as the analytical expanded uncertainty of the official method (U ≈ 132 mg/kg; ^†^ evaluated at the 1000 mg/kg decision limit, k = 2), indicating that the contribution of secondary biological factors to total-sterol variance, beyond measurement uncertainty, is small.

**Table 3:** Main factors influencing phytosterol composition and interpretation in Greek olive oil.

Factor	Expected Influence on Sterolic Profile	Strength of Evidence in Greek Olive Oil	Main Interpretative Implication	[Ref.]
Cultivar/genetic background	Primary determinant of total sterols and relative sterol distribution. Koroneiki tends to show lower total sterol values compared with other Greek cultivars.	Strong for Koroneiki, Mastoides and *Lianolia Kerkyras*; limited for many other Greek cultivars.	Sterol values should first be interpreted against cultivar-specific baselines.	[15,16,18,20]
Geographical origin/terroir	May cause secondary quantitative shifts in the overall chemical fingerprint; direct sterol-specific evidence remains limited.	Moderate for broader Greek EVOO compositional fingerprints; limited for sterol-specific geographical effects.	Geography should refine, not redefine, the cultivar-dependent sterolic profile.	[6,7,14,19]
Harvest maturity	May gradually affect total sterols and relative proportions of individual sterols and triterpene diols.	Well supported in broader olive-oil literature; limited Greek sterol-specific maturity datasets.	Maturity should be treated as a secondary modulator, especially in cultivar-controlled interpretation.	[20]
Processing conditions	Generally limited direct effect under standard EVOO mechanical extraction; possible indirect effects through oxidation or storage environment.	Limited Greek sterol-specific evidence; stronger evidence for effects on phenolics, volatiles and oxidative stability.	Processing effects should be interpreted cautiously unless extreme conditions or refining are involved.	[20,24,53]
Storage/oxidation	Phytosterols are relatively stable but may undergo oxidation under adverse light, oxygen and temperature conditions.	Limited Greek sterol-specific storage evidence.	Sterols are robust markers but not chemically inert; sterol oxidation products represent a future research gap.	[8,20,53]
Analytical methodology/reporting	Sample preparation, chromatographic separation, peak integration and percentage-based reporting may affect near-threshold interpretation.	Strongly relevant due to official EU/IOC workflows; limited Greek inter-method comparison studies.	Methodological transparency is essential when values lie close to regulatory limits.	[8,22,25,44]

**Table 4:** Regulatory decision outcomes for a sterolic result under the Near-Threshold Sterolic Space framework.

Measured Value *X* vs. Guard Band	Consistent with Cultivar Reference Profile?	Regulatory Decision Outcome	Action
*X* > *L* + *U*	—	Compliant	Routine acceptance; parameter resolved
*X* < *L* − *U*	—	Non-compliant	Routine flag/rejection; parameter resolved
*L* − *U* ≤ *X* ≤ *L* + *U* (NTSS)	Yes	Conditional pass—authentic near-threshold oil	Accept under conditional and complementary assessment; apply any cultivar-specific derogation in force
*L* − *U* ≤ *X* ≤ *L* + *U* (NTSS)	No	Conditional fail/suspect	Refer to further authentication before a decision

## Data Availability

No new experimental data were generated in this study. All data discussed are available in the cited published literature. The datasets compiled and re-analyzed to produce Figure 1 and Figure 2—together with their sources, sample sizes, crop years, geographical origins and analytical methods—are provided in full in Supplementary Table S1.

## Supplementary Materials

The following supporting information can be downloaded at: https://www.mdpi.com/article/10.3390/molecules31183170/s1. Table S1: Source data underlying Figure 1 and Figure 2: reported phytosterol composition of the Greek monovarietal extra virgin olive oils compiled in this review, with sample size, crop year, geographical origin, analytical method and reference for each dataset. Table S2: Literature search string and parameters used to assemble the evidence base of this review.

## Abbreviations

The following abbreviations are used in this manuscript:

ASGs	Acyl Steryl Glycosides
EU	European Union
EVOO	Extra Virgin Olive Oil
FSs	Free Sterols
GC-FID	Gas Chromatography-Flame Ionization Detector
GC-MS	Gas Chromatography-Mass Spectrometry
IOC	International Olive Council
ISO	International Organization for Standardization
IUPAC	International Union of Pure and Applied Chemistry
k	Coverage Factor
ML	Machine Learning
NGS	Next-Generation Sequencing
NMR	Nuclear Magnetic Resonance
NTSS	Near-Threshold Sterolic Space.
PCA	Principal Component Analysis
PDO	Protected Designation of Origin
PLS	Partial Least Squares
RSD_R_	Relative Reproducibility Standard Deviation
SD	Standard Deviation
SD_R_	Reproducibility Standard Deviation
SEs	Steryl Esters
SGs	Steryl Glycosides
U	Expanded (Measurement) Uncertainty
VOO	Virgin Olive Oil
