# Supplementary material for: Beyond Phytosterol Profiling in Greek Olive Oil: Biological Variability, Regulatory Interpretation and the Near-Threshold Sterolic Space
Source: Molecules. 2026 Sep 9;31(18):3170. doi: 10.3390/molecules31183170 (PMC13609128; doi:10.3390/molecules31183170)
Supplement: Supplementary file 1 [file molecules-31-03170-s001.zip › Table S1.pdf]

Supplementary Materials

Table S1. Source data underlying Figures 1 and 2: reported phytosterol composition of the Greek monovarietal extra virgin olive oils compiled in this review, with sample size, crop year, geographical origin, analytical method and reference for each dataset.

Parameter	Mean ± SD	Range (min–max)	Notes
cv. Koroneiki -- Messinia (SW Peloponnese, Greece) 2014–2015 crop year N = 69 GC-FID per Reg. (EEC) 2568/91 (≡ IOC COI/T.20/Doc. No 26) ISO 17025 accredited lab Source: Skiada et al. 2019 [1]; same dataset in Skiada et al. 2020 [2]			
Total sterols (mg/kg)	1033.3 ± 150.1	744–1283	43.5% of samples < 1000 mg/kg (EEC); 66.7% < 1100 mg/kg (Kalamata PDO). Near-threshold parameter.
Campesterol (%)	3.71 ± 0.38	2.78–4.70	21.7% of samples exceeded 4.0%. Near-threshold parameter.
β-Sitosterol (%)	80.73 ± 3.73	--	Predominant sterol.
Δ^5-Avenasterol (%)	12.28 ± 3.96	--	
Stigmasterol (%)	0.74 ± 0.19	--	
Apparent β-sitosterol (%)	94.63 ± 1.07	--	Limit ≥ 93.0%; compliant.
Δ^7-Stigmastenol (%)	0.19 ± 0.09	--	Limit ≤ 0.5%; compliant.
Cholesterol (%)	0.11 ± 0.03	--	
24-Methylene-cholesterol (%)	0.32 ± 0.09	--	
Campestanol (%)	0.05 ± 0.03	--	< campesterol; compliant.
Clerosterol (%)	0.85 ± 0.07	--	
Sitostanol (%)	0.37 ± 0.30	--	
$\Delta^{5,24}$-Stigmastadienol (%)	0.29 ± 0.10	--	
Δ^7-Avenasterol (%)	0.28 ± 0.11	--	
Total erythrodiol + uvaol (%)	2.85 ± 1.25	--	Limit ≤ 4.5%; 8.06% of samples exceeded 4.5%.
cv. Mastoides (Athinolia) - SE Lakonia (S Peloponnese, Greece) 2014-2015 crop year N = 43 GC-FID per Reg. (EEC) 2568/91 (≡ IOC COI/T.20/Doc. No 26) ISO 17025 accredited lab Source: Skiada et al. 2020 [15]			
Total sterols (mg/kg)	1219.6 ± 109.2	--	Comfortably above 1000 mg/kg.
Campesterol (%)	3.14 ± 0.16	--	
β-Sitosterol (%)	84.12 ± 2.69	--	Higher than Koroneiki ($p < 0.05$).
Δ^5-Avenasterol (%)	9.85 ± 2.66	--	Lower than Koroneiki ($p = 0.001$).
Stigmasterol (%)	0.64 ± 0.18	--	
Apparent β-sitosterol (%)	95.45 ± 0.29	--	Limit ≥ 93.0%; compliant.
Δ^7-Stigmastenol (%)	0.18 ± 0.09	--	Limit ≤ 0.5%; compliant.
Cholesterol (%)	0.12 ± 0.03	--	
24-Methylene-cholesterol (%)	0.19 ± 0.05	--	

Parameter	Mean ± SD	Range (min–max)	Notes
Campestanol (%)	0.04 ± 0.02	--	
Clerosterol (%)	0.94 ± 0.07	--	
Sitostanol (%)	0.31 ± 0.08	--	
$\Delta^{5,24}$-Stigmastadienol (%)	0.22 ± 0.06	--	
Δ^{7}-Avenasterol (%)	0.22 ± 0.06	--	
Total erythrodiol + uvaol (%)	1.40 ± 0.52	--	Limit ≤ 4.5%; compliant.
cv. Koroneiki -- coastline of NW Greece (Preveza / Parga / Thesprotia) 2019–2020 crop year N = 44 GC-FID per Reg. (EEC) 2568/91 (≡ IOC COI/T.20/Doc. No 26) ISO 17025 accredited lab Source: Skiada et al. 2020 [16]			
Total sterols (mg/kg)	1020.8 ± 120.7	--	40.9% of samples < 1000 mg/kg. Near-threshold parameter; independently reproduces the Messinia Koroneiki pattern.
Campesterol (%)	3.82 ± 0.35	--	Near-threshold parameter.
β-Sitosterol (%)	85.95 ± 2.68	--	
Δ^{5}-Avenasterol (%)	6.93 ± 2.38	--	
Stigmasterol (%)	0.63 ± 0.18	--	
Apparent β-sitosterol (%)	94.63 ± 0.70	--	Limit ≥ 93.0%; compliant.
Δ^{7}-Stigmastenol (%)	0.32 ± 0.15	--	Limit ≤ 0.5%; compliant.
Cholesterol (%)	0.10 ± 0.08	--	
24-Methylene-cholesterol (%)	0.23 ± 0.09	--	
Campestanol (%)	0.07 ± 0.03	--	
Clerosterol (%)	0.81 ± 0.20	--	
Sitostanol (%)	0.48 ± 0.24	--	
$\Delta^{5,24}$-Stigmastadienol (%)	0.29 ± 0.14	--	
Δ^{7}-Avenasterol (%)	0.25 ± 0.16	--	
Total erythrodiol + uvaol (%)	2.76 ± 1.07	--	Limit ≤ 4.5%; compliant.
cv. Lianolia Kerkyras -- coastline of NW Greece (Preveza / Parga / Thesprotia) 2019–2020 crop year N = 60 GC-FID per Reg. (EEC) 2568/91 (≡ IOC COI/T.20/Doc. No 26) ISO 17025 accredited lab Source: Skiada et al. 2020 [16]			
Total sterols (mg/kg)	1343.7 ± 115.1	--	Comfortably above 1000 mg/kg.
Campesterol (%)	3.42 ± 0.17	--	
β-Sitosterol (%)	89.21 ± 1.27	--	Highest β-sitosterol of the four datasets.
Δ^{5}-Avenasterol (%)	4.31 ± 1.27	--	Lowest Δ^{5}-avenasterol of the four datasets.
Stigmasterol (%)	0.49 ± 0.15	--	
Apparent β-sitosterol (%)	95.28 ± 0.35	--	Limit ≥ 93.0%; compliant.

Parameter	Mean ± SD	Range (min–max)	Notes
Δ^7-Stigmastenol (%)	0.29 ± 0.11	--	Limit ≤ 0.5%; compliant.
Cholesterol (%)	0.12 ± 0.06	--	
24-Methylene-cholesterol (%)	0.08 ± 0.04	--	
Campestanol (%)	0.04 ± 0.02	--	
Clerosterol (%)	0.81 ± 0.16	--	
Sitostanol (%)	0.69 ± 0.17	--	
$\Delta^{5,24}$-Stigmastadienol (%)	0.22 ± 0.11	--	
Δ^7-Avenasterol (%)	0.26 ± 0.11	--	
Total erythrodiol + uvaol (%)	1.43 ± 0.45	--	Limit ≤ 4.5%; compliant.

Notes on data compilation and weighting. Values are the study-reported means ± standard deviation of independent olive-oil samples; ranges (min–max) are given where reported in the source. All datasets were generated in a single ISO 17025-accredited laboratory by capillary GC-FID following the official EU/IOC procedure (Reg. (EEC) 2568/91, Annex V, later amendments; equivalent to IOC COI/T.20/Doc. No 26), with α-cholestanol as internal standard. Individual sterols are expressed as percentages of total sterols and total sterols in mg/kg of oil. No meta-analytic weighting was applied: each horizontal bar in Figure 1 and each proportion in Figure 2 corresponds to an individual study's reported mean (and range, where available) rather than to a pooled or sample-size-weighted estimate. The two Koroneiki datasets are independent (different regions and crop years) and are shown separately; their close agreement for total sterols (1033.3 and 1020.8 mg/kg) is treated as reproducibility evidence, not as replication of a single dataset. The ranges shown here are between-sample (biological) dispersion and should not be confused with the analytical measurement uncertainty of the method, which is discussed separately in the main text.

Abbreviations: *GC-FID, gas chromatography with flame-ionisation detection; EEC/EU, European Union; IOC, International Olive Council; SD, standard deviation; N, number of samples.*

References

1. Skiada, V.; Tsarouhas, P.; Varzakas, T., Preliminary study and observation of "kalamata PDO" extra virgin olive oil, in the messinia region, southwest of peloponnese (Greece). *Foods* **2019,** 8, (12).
2. Skiada, V.; Tsarouhas, P.; Varzakas, T., Comparison and discrimination of two major monocultivar extra virgin olive oils in the southern region of Peloponnese, according to specific compositional/traceability markers. *Foods* **2020,** 9, (2).
