# Supplementary material for: Beyond Phytosterol Profiling in Greek Olive Oil: Biological Variability, Regulatory Interpretation and the Near-Threshold Sterolic Space
Source: Molecules. 2026 Sep 9;31(18):3170. doi: 10.3390/molecules31183170 (PMC13609128; doi:10.3390/molecules31183170)
Supplement: Supplementary file 1 [file molecules-31-03170-s001.zip › Table S2.pdf]

Supplementary Materials

Table S2. Literature search string and parameters used to assemble the evidence base of this review.

Parameter	Detail
**Database**	Scopus (Advanced Search)
**Search date**	4 August 2026
**Publication window**	2010–2026 (`PUBYEAR > 2009 AND PUBYEAR < 2027`)
**Language**	English
**Document types**	Article; Review
**Search field**	`TITLE-ABS-KEY`
**Records retrieved**	37 (0 duplicates)
**Excluded on screening**	5
**Included (evidence base)**	32
**Search string**	`(TITLE-ABS-KEY (sterol* OR phytosterol* OR "beta-sitosterol" OR campesterol OR avenasterol OR stigmasterol OR stigmastenol OR "triterpene dialcohol*" OR erythrodiol OR uvaol)) AND (TITLE-ABS-KEY ("olive oil" OR EVOO OR "virgin olive oil" OR "Olea europaea")) AND (TITLE-ABS-KEY (Greece OR Greek OR Koroneiki OR Mastoides OR Athinolia OR "Lianolia Kerkyras" OR Crete OR Cretan OR Peloponnese OR Messinia OR Kalamata OR Zakynthos OR Lesvos OR Kolovi OR Adramytini OR Throumbolia OR Manaki)) AND PUBYEAR > 2009 AND PUBYEAR < 2027`

Note. Search performed in Scopus (Advanced Search) on 4 August 2026. The query combined three concept blocks — sterols/phytosterols, olive oil, and Greek origin or cultivar — with Boolean operators; an asterisk (*) denotes truncation and quotation marks denote exact phrases. Results were limited to English-language articles and reviews published between 2010 and 2026. Of 37 records retrieved (no duplicates), 5 were excluded (two non-olive matrices; one clinical review without compositional data; one lacking extractable sterol data; one conference paper) and 32 were retained as the empirical evidence base; additional methodological, regulatory and foundational sources were identified through citation tracking of the retrieved articles and of the official EU/IOC documents.
